# Dynamische Entwicklung der COVID-19-Intensivbelegung im Herbst/Winter 2021/22 in Abhängigkeit von den 7-Tage-Inzidenzen

**DOI:** 10.1007/s00063-022-00904-w

**Published:** 2022-02-09

**Authors:** Andreas Schuppert, Christian Karagiannidis

**Affiliations:** 1 Institut für Computational Biomedicine, JRC für Computational Biomedicine, Universitätsklinikum Aachen, RWTH Aachen University, Aachen, Deutschland; 2 Lungenklinik Köln-Merheim, ARDS und ECMO Zentrum, Kliniken der Stadt Köln, Witten/Herdecke Universitätsklinikum, Köln, Deutschland

**Keywords:** Belastungsmodell, Modellierung, Szenario, Überlastung, Intensivstation, Load model, Modeling, Scenario, Overload, ICU

Trotz zunehmender Impferfolge mit mittlerweile mindestens 66 % vollständig immunisierten Personen in Deutschland nach Angaben des Robert Koch-Instituts ist es im Herbst 2021 zu einem erneuten Anstieg der Infektionszahlen mit SARS-CoV‑2 gekommen. Vielfach wird die Frage gestellt, wie dieses bei derart hohen Impfquoten möglich ist und insbesondere auch wie stark sich die Inzidenzen auf die intensivstationäre Belastung auswirken.

Bei den aktuellen Impfquoten nach RKI-Impfquotenmonitoring und Berücksichtigung der Genesenen entsprechend RKI-Dashboard erwarten wir 68 Mio. Immunisierte und 15,8 Mio. suszeptible Menschen in Deutschland, verteilt auf die Altersgruppen 0–17, 18–59 und 60+. Aus den DIVI-Neuaufnahmedaten können wir mit einem aktuellen Risikoprofil für eine Aufnahme auf die ITS bei Infektion je nach Altersgruppe zwischen 0 und 4,5 % (Tab. 1) rechnen.

**Table 1:** 

	0–17	18–59	60+
Immunisiert	0	0,1 %	1,57 %
Suszeptibel	0,07 %	1,68 %	4,49 %

Diese Werte sind momentan mit einer Unsicherheit von etwa 10 % behaftet. Mit dieser Risikoverteilung auf die Bevölkerungskohorten erhalten wir dann unter Annahme einer vollständigen Infektion/Immunisierung der Bevölkerung bei den aktuellen Impfquoten noch etwa 270.000 Patienten, die potenziell intensivpflichtig werden können mit einer durchschnittlichen Belegungsdauer der Intensivstation von aktuell etwa 20 Tagen. Diese 270.000 setzen sich zusammen aus 38.000 Geimpften oder Genesenen, die trotzdem aufgrund ihrer Grunderkrankungen, des Alters oder verminderten Impfansprechens intensivpflichtig werden können, sowie 232.000 Nichtgeimpften. Es sei aber bemerkt, dass hier die zeitliche Verteilung des Auftretens der Erkrankung in den kommenden Monaten und Jahren von entscheidender Bedeutung ist ebenso wie das Eintreten der Herdenimmunität, bevor alle potenziell Intensivpflichtigen auch wirklich intensivpflichtig werden.

**Figure 1:**
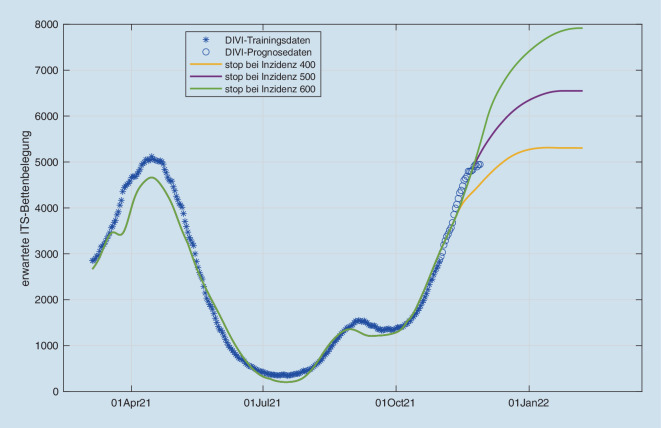

**Figure 2:**
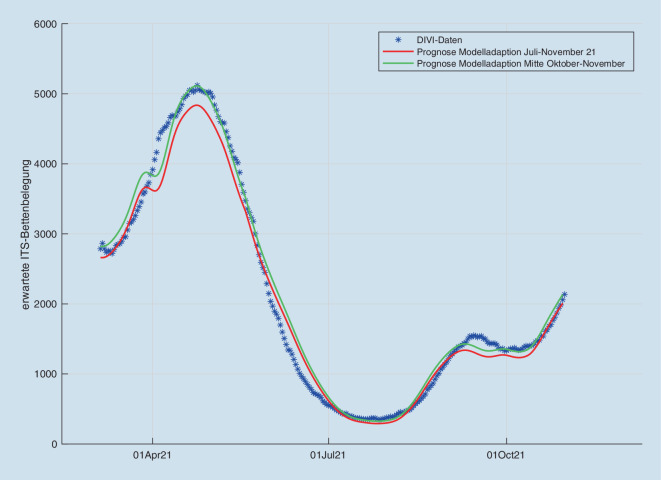

Zur Vorhersage der weiteren Intensivbettenbelastung haben wir unsere Modelle [3, 4] um die aktuellen Impfquoten, sowohl Grundimmunisierung als auch Auffrischungsimpfungen, Waning-Effekte (nachlassende Impfwirkung) und die Infektionsdynamik der Delta-Welle in der näheren Zukunft erweitert (Abb. 1). In den simulierten Szenarien gehen wir von einer Fortsetzung des momentan beobachteten Trends aus, mithilfe eines polynomialen Schätzers quantifiziert wird. Da bei kritischen Infektionslagen sowohl staatliche Regelungen als auch individuelle Vorsichtsmaßnahmen mit schwer a priori quantifizierbaren Effekten zu erwarten sind [1], simulieren wir den momentanen Trend bis zu Inzidenzen von 400−600, danach nehmen wir jeweils eine auf R = 1 geregelte stationäre Infektionsdynamik an. Die Inzidenzen und ihr Anstieg hier spiegeln sich auch weiterhin in der Intensivbelegung wider, allerdings ist der Anstieg nicht ganz so stark ausgeprägt wie in den Wellen 1−3. In Abhängigkeit von den Inzidenzen erwarten wir erneut jedoch mindestens 5000 COVID-19-Patienten auf den Intensivstationen, aufgrund der Impfquoten aber mit erheblichen regionalen Unterschieden. Die erwartete Intensivbelegung wird in Abb. 1 gezeigt, jeweils mit eine STOP-Szenario auf dem jeweiligen Inzidenzniveau. Die Risiken für eine intensivpflichtige Infektion wurden mit unserem Modell anhand der Intensivregisterintensivaufnahmedaten (www.intenisvregister.de) und der altersstratifizierten Inzidenzen des RKI-Dashboards geschätzt. Hierzu wurden die Risikoparameter einerseits an den Daten im Intervall 04.07.2021 bis 15.11.2021 sowie im Intervall 18.10.2021 bis 15.11.2021 durch Maximum-Likelihood-Schätzer bestimmt. Dabei zeigte sich eine strukturelle Diskrepanz zwischen beiden Schätzern, die sich durch eine angenommene saisonale Abschwächung des angenommenen Impfschutzes gegen Infektion, nicht aber gegen schwere Erkrankung, sowie die vermehrte Anwendung von 2G-Regelungen, kurieren ließ. Abb. 2 zeigt, dass damit überraschenderweise der anhand der letzten 14 Tage identifizierte Schätzer einen sehr guten Fit auch außerhalb des jeweiligen Trainingsbereichs schon ab April 2021 zeigt, was die Modellannahmen unterstützt. Mit der Annahme eines Waning-Effekts allein ca. 6 Monaten nach Impfung hingegen konnte keine befriedigende Koinzidenz zwischen kurz- und langfristigem Schätzer erreicht werden. Eine Erklärung könnte sein, dass vermehrte Infektionen in Innenräumen tendenziell mit einer höheren Viruslast einhergehen, die ein höheres Risiko einer nachweisbaren Infektion mit mildem Verlauf bewirkt, bevor die durch Impfung induzierte Immunantwort einsetzt. Ebenso könnte das Verhalten durch eine verstärkte Nutzung von 2G-Regelungen erklärt werden, die das im Modell als gleichverteilt angenommene Infektionsrisiko von den nichtgeimpften Bevölkerungsgruppen zu den geimpften Bevölkerungsgruppen hin verschiebt. Weiterhin sind die beschriebenen Waning-Effekte aus Israel zum Teil auch auf die im Vergleich zu Deutschland kürzeren Impfabstände zurückzuführen [2].

Die entscheidende Frage wird sein, ob und wie der Aufwärtstrend gestoppt werden kann. Dies kann zum einen darin bestehen, dass Booster-Impfungen breitflächig ausgerollt werden und damit einen substanziellen Einfluss auf den R‑Wert bekommen, Kontaktbeschränkungen für Ungeimpfte in Form von 2G kommen könnten oder insbesondere auch dass die Bevölkerung freiwillig ihr Kontaktverhalten verändert, weil die Infektionszahlen irgendwann so hoch sind, dass ein Umdenken stattfindet. Letzteres Szenario würde dazu führen, dass wir auf einem bestimmten Infektionsniveau verbleiben und sich letzten Endes bis voraussichtlich April/Mai 2022 ein Plateau einstellt. Aus Sicht der intensivmedizinischen Kapazitäten wäre dies eine gangbare Lösungsmöglichkeit, weil letzten Endes ein Steady State der COVID19-Patienten auf den Intensivstationen resultiert und sich die Kliniken entsprechend anpassen könnten. In Anbetracht der großen noch potenziell intensivpflichtig werdenden Patientenzahl könnte dies eine wesentliche Lösungsmöglichkeit sein, um durch die kommenden Jahre zu kommen. Wenn von Jahr zu Jahr der Anstieg der Inzidenzen und damit auch die damit verbundene Intensivbelastung geringer ausfällt, könnte die Coronapandemie wie eine Schwingung, die von Saison zu Saison geringer wird, auslaufen. Hierzu wäre es aber essenziell, dass die Inzidenzen nur so weit steigen, dass dies intensivmedizinisch auch noch geleistet werden kann.
